# Severe Hyperthyroidism Masquerading as Acute Bulbar Weakness

**DOI:** 10.7759/cureus.1716

**Published:** 2017-09-27

**Authors:** Rena D Sukhdeo, Christopher Jackson, Cyrilyn Walters, Aneel Kumar

**Affiliations:** 1 Neurology, University of Tennessee Health Science Center Memphis; 2 Internal Medicine, University of Tennessee Health Science Center Memphis

**Keywords:** hyperthryoidism, stroke, weakness, bulbar, endocrine, mimic, tia, cva, hypothyroidism

## Abstract

The stroke occurs in nearly 800,000 patients per year in the United States with significant morbidity if not treated and managed in a time sensitive manner. Acute bulbar weakness can be a common presenting manifestation of acute stroke and transient ischemic attack. However, up to 30% of the patients presenting with symptoms concerning for stroke could be displaying a disease process that mimics the stroke. These disorders include hypoglycemia, seizures, complicated migraines, and endocrinopathies such as thyroid disease. If neuroimaging fails to show evidence of an acute infarct, these stroke mimics should be considered. When acute bulbar weakness occurs in the setting of severe hyperthyroidism, the treatment of this process can result in rapid improvement of symptoms.

## Introduction

Acute bulbar weakness can be a common presentation of a cerebrovascular accident (CVA) or transient ischemic attack (TIA). However, other etiologies to consider include idiopathic/ virus-induced pathologies such as Bells palsy; botulism, myasthenia gravis, hypoglycemia, complicated migraine, and endocrinopathies such as thyroid disease. These processes are generally called stroke mimics because they present with features that imitate an acute vascular event. Endocrinopathies are a rare precipitant of acute bulbar weakness, however when patients present with thyroid-related weakness, the disease association is has been seen in hypothyroidism with myopathy. We present a case of acute bulbar weakness mimicking a stroke in a patient with severe hyperthyroidism.

## Case presentation

A 73-year-old right-handed African-American female with a past medical history of paroxysmal atrial fibrillation, Graves’ disease, and essential hypertension presented to the emergency room after the sudden onset of dysarthria and dysphagia six hours prior to the admission. Her dysarthria was marked such that she could not speak one word without significant difficulty. Her dysphagia was noted to solids and liquids when she attempted to drink water and eat chicken at a social function. The patient denied lateralizing weakness, vision loss, confusion, bladder/bowel incontinence, dystonia, or previous episodes of weakness. She did not have a previous history of cerebrovascular accident (CVA) or transient ischemic attack (TIA). She denied a history of recent head trauma, major surgery, gastrointestinal bleeding, bleeding diathesis, or known neurologic disease. The patient was taking methimazole for Graves’s disease, but four weeks prior to admission, it was held for a radioactive iodine and uptake scan. However, the patient was restarted on a reduced methimazole dose due to increased tremulousness, anxiety, and diarrhea two weeks prior to her presentation in our emergency room. The patient’s initial vital signs were significant for tachycardia up to 116 beats per minute and tachypnea up to 24 breaths per minute, with a presenting blood pressure of 142/84. The physical examination, including a full neurologic exam, revealed only moderate dysarthria with stuttering. The patient’s sensorium, cranial nerve examination, strength, and sensation were intact with no focal weakness, sensory deficits, or cerebellar signs. The National Institutes of Health Stroke Scale (NIHSS) score for the patient was three due to her speech difficulties; the airway, breathing, circulation, disability (ABCD) scores were five, and her premorbid Modified Rankin Score was zero. The laboratory findings on admission were significant for a thyroid stimulating hormone (TSH) level of 0.01 µIU/mL, free T4 of 4.43 ng/dL, and thyroid stimulating immunoglobulins of 537. Electrocardiogram showed sinus tachycardia with a rate of 116 beats per minute, normal axis, normal PR and QT intervals, and no ST segment or T wave changes. Computed tomography of the head (CTH) without contrast was negative for hemorrhage. She was admitted to the hospital for concern of CVA versus TIA. The patient did not receive intravenous tissue plasminogen activator (tPA) due to the duration of her symptoms as well as her low NIHSS score. Magnetic resonance imaging of the brain was negative for acute ischemic change or space-occupying lesion. Computed tomography angiography (CTA) of the head and neck demonstrated no significant intracranial arterial disease. Stroke laboratory evaluation including vitamin B12, homocysteine, human immunodeficiency virus and rapid plasma reagin were all unremarkable. Serial neurologic exams failed to show new or evolving neurological deficits. Despite her negative evaluation, her dysarthria persisted for more than 24 hours after initial hospitalization. Additionally, oropharyngeal dysphagia was appreciated on bedside swallow evaluation by speech therapy. Given the findings on initial laboratory studies, endocrinology was consulted for further recommendations. They increased her methimazole dose and started Propranolol. Within 48 hours of adjusting her anti-hyperthyroid medications, the patient’s dysarthria and dysphagia rapidly improved. Videofluoroscopy on day three of hospitalization showed no evidence of oropharyngeal dysphagia. The patient was discharged on 81 mg aspirin and 40 mg Atorvastatin with the follow-up to endocrinology and neurology as an outpatient. During the outpatient neurology evaluation, the patient continued to be free of dysarthria, dysphagia, and the stigmata of TIA or CVA.

## Discussion

In the patients with acute onset bulbar weakness, stroke remains an important differential diagnosis. It is not just a crucial parameter for deciding if a patient can get tPA, but also an important quality measure for physicians trying to save the tissue. A cohort study among eight Canadian emergency rooms revealed a prominent correlation between acutely isolated dysarthria with bulbar weakness and stroke [1]. Thus, patients presenting with symptoms of rapid onset bulbar weakness and dysarthria that are suspected to be having a vascular event, are typically managed according to the high-risk TIA guidelines. However, approximately nine to 30% of the patients with a presumptive diagnosis of stroke and up to 17% of the patients who received tPA are displaying clinical symptoms of another process that mimics a stroke [2]. Disorders such as hypoglycemia, seizures with the post-ictal state, complicated migraines, myasthenia gravis, acute viral illness, and endocrinopathies such as hypo- or hyper-thyroidism can all present with stroke-like features such as dysarthria, bulbar weakness, or even hemiparesis [3-4].

Thyroid hormone abnormalities are a rare precipitant of bulbar weakness and dysarthria. In a literature review done in 2004 by Chiu, et al. [5], severe thyrotoxicosis and thyroid storm rarely precipitated dysarthria and dysphagia. However, their review found that the patients with thyrotoxicosis usually have some degree of thyrotoxic myopathy or neuropathy preceding the development of dysarthria. The associated myopathy has been documented to be in the proximal limbs and minimally improves the management of the thyroid dysfunction. In one case report, a middle-aged female presented with isolated dysarthria, associated with hypothyroidism, that responded to appropriate hormone replacement therapy [6]. Including that case report, just one other instance was documented about hypothyroidism and dysarthria. In this specific case, it appeared that cause of the dysarthria was edema of the larynx and associated structures. In general, the recovery from the acute process of bulbar weakness, dysarthria, and dysphagia can be abated with the initiation of the thyroid modulating medications. However, recovery is protracted, often taking weeks to months of hormone replacement therapy to become euthyroid. In our case, the patient presented with acute onset bulbar weakness, with dysarthria and oropharyngeal dysphagia. Our patient had no signs or symptoms of thyrotoxic myopathy and never described muscle weakness or pain in her proximal limbs. On videofluoroscopy, no edema of the larynx or tongue was visualized either, as it was in the case report of hypothyroidism-associated dysphagia. Our patient demonstrated marked improvement with the initiation of the appropriate dosing of thyroid mediating treatment.

Often, thyroid hormone abnormalities are not usually the first differential, when considering processes that mimic a stroke. The initial work-up for a person presenting with new bulbar weakness includes serum glucose level, NIHSS score assessment, orientation questioning, electrocardiogram (EKG), CTH, and/or CTA. Once these tests are performed, if clinically indicated, tPA is administered. The rest of the laboratory values that are ordered include TSH, homocysteine, rapid plasma reagin (RPR) test, human immunodeficiency virus (HIV) test, Anti-neutrophil cytoplasmic antibodies (ANCA), anti-nuclear antibody (ANA), and other blood tests depend on the patient-specific features. The utility of measuring TSH is often overlooked in suspected acute CVA. However, in scenarios such as the case we presented, ordering a TSH, was an essential part of diagnosing the cause of the defect in our patient. The rapid recovery of our patient suggests that thyrotoxic-mediated bulbar weakness may represent a clinical spectrum, in which early intervention can hasten recovery and reduce morbidity from this syndrome.

## Conclusions

Acute bulbar weakness can be a sign of acute CVA or TIA, and therefore, these patients should be treated accordingly. However, when neuroimaging fails to demonstrate evidence of acute CVA, the patients should be aggressively evaluated for another process masquerading as stroke. While rare, hyperthyroidism can mimic acute stroke, and quick treatment can result in rapid improvement of observed neurologic symptoms.

## References

[1] Beliavsky A, Perry JJ, Dowlatshahi D (2014). Acute isolated dysarthria is associated with a high risk of stroke. Cerebrovasc Dis Extra.

[2] Libman RB, Wirkowski E, Alvir R (1995). Conditions that mimic stroke in the emergency department. Implications for acute stroke trials. Arch Neurol.

[3] Kamalian S, Kamalian S, Boulter DJ (2015). Stroke differential diagnosis and mimics: Part 1. Appl Radiol.

[4] Turner M, Tablot K (2017). Mimics and chameleons in motor neuron disease. Pract Neurol.

[5] Chiu WY, Yang CC, Huang I (2004). Dysphagia as a manifestation of thyrotoxicosis: report of three cases and literature review. Dysphagia.

[6] El-Shafie KT (2003). Hypothyroidism presenting with dysarthria. J Family Community Med.

